# Effect of peripheral nerve block versus general anesthesia on the hemodynamics and prognosis of diabetic patients undergoing diabetic foot Surgery

**DOI:** 10.1186/s13098-023-01185-9

**Published:** 2023-10-26

**Authors:** Gehua Zhu, Jiamin Xu, Hanying Dai, Dinghong Min, Guanghua Guo

**Affiliations:** Medical Center of Burn Plastic and Wound Repair, The First Affiliated Hospital of Nanchang University, Nanchang, China

**Keywords:** Diabetic foot, Hemodynamic stability, Popliteal nerve block, Postoperative complication, Postoperative pain

## Abstract

**Background:**

Diabetic foot ulcers (DFUs) represent a significant foot-related concern for patients with multiple co-morbidities, and surgical intervention is often employed. Notably, peripheral nerve block anesthesia (PNB) has emerged as a new approach for the surgical management of DFUs, providing sustained hemodynamic stability and superior postoperative pain control compared to general anesthesia (GEA).

**Methods:**

The present study utilized a retrospective analysis of hospitalized patients who met the inclusion criteria for DFUs over a period of 7 years. Patients were categorized into two groups based on the type of anesthesia employed during the procedure: GEA or PNB. Extensive patient information was gathered and analyzed, such as demographics, intraoperative hemodynamic parameters, numeric rating scale (NRS) scores, and healing outcomes. The preliminary results assessed in this study were intraoperative hemodynamic stability and postoperative analgesic efficacy.

**Results:**

During the study period, 117 patients received surgical therapy based on GEA, while 145 patients received PNB. Notably, the mean intraoperative blood pressure was significantly lower in the GEA group, and this difference remained statistically significant even after Bonferroni adjustment using linear mixed models. Additionally, the frequency of hypotensive episodes was higher in the GEA group (P < 0.05). Furthermore, the perioperative transfusion volume, overall intraoperative fluid input, and intraoperative bleeding volume were significantly more significant in the GEA group than in the PNB group. The postoperative pain NRS scores differed considerably between the two groups (Bonferroni corrected P < 0.01), with the GEA group exhibiting higher opioid consumption on the day of surgery and the first postoperative day when using patient-controlled intravenous analgesia (PCIA). Supplemental analgesic medication was more significant in the GEA group 24 h postoperatively. However, the two groups had no difference in hospital stay or treatment outcomes. There was no difference between the two groups regarding secondary surgery and amputation procedures. Although the 5-year mortality rate is 30.5%, no significant difference in mortality rates between the two groups was observed.

**Conclusions:**

Compared to GEA, PNB is a safe and effective alternative therapy for managing DFUs. Our findings suggest that PNB administration during surgical intervention for this condition results in more stable intraoperative hemodynamics and superior postoperative analgesic effects, despite no significant difference in overall treatment outcomes between the two groups. The two groups did not differ in re-surgery, amputation, or 5-year mortality.

**Supplementary Information:**

The online version contains supplementary material available at 10.1186/s13098-023-01185-9.

## Introduction

Diabetes mellitus significantly impairs global public health, with over 422 million individuals affected worldwide, according to 2014 statistics [1]. This trend is expected to continue, with an estimated 642 million individuals projected to live with diabetes over the next two decades [2]. The challenging course of therapy, high medical costs, and increased incidence burden patients’ financial and mental well-being [3], particularly for diabetic foot, one of the most severe complications of diabetes. Peripheral neuropathy, peripheral artery disease, and foot ulcers are common ailments among lower-limb diabetics. Research shows that 10–25% of diabetic individuals will develop DFUs at some point [4, 5], within five years of ulcer onset, 5% of individuals will undergo limb amputation, while 50-70% are expected to experience mortality [6–8]. The conventional approach for treating this condition entails surgical debridement or amputation. However, recent studies have demonstrated that multiple debridement procedures can significantly enhance wound healing rates and thus represent a valuable therapeutic option [9]. However, most patients with DFUs are older, have had diabetes for an extended period, have at least one comorbid condition, and suffer from multiple organ disorders [10–13]. Ensuring stable hemodynamics while administering appropriate anesthetic and analgesia is a critical concern. PNB with ultrasound guidance have garnered increased acceptance stemming from their heightened efficacy and capacity to ameliorate systemic complications associated with GEA. In contrast to the adverse impact of the latter on cardiopulmonary function, ultrasound-guided PNB have proved instrumental in not only impeding the activation of inflammatory factors [14, 15] but also in restraining the physiological reaction engendered by surgical trauma, thereby curtailing the accompanying injury. In this retrospective study, we examined the impact of GEA and PNB anesthetic techniques on intraoperative hemodynamics, postoperative analgesia, and treatment results for DFUs.

## Materials and methods

This study was approved by the Ethical Review Committee of the First Affiliated Hospital of Nanchang University, and the subjects’ written consent in each case was obtained. Ethics number of this study: MR-36-23-015232.

To draw definitive conclusions, this scientific article examines clinical data obtained from patients who underwent surgical treatment for DFUs at our hospital over seven years.

The inclusion criteria for our study included patients aged 18 years and above, of any gender, who underwent surgery for DFUs and received either GEA or ultrasound-guided PNB. Exclusion criteria comprised patients with severe amputations above the ankle, incomplete clinical data (missing preoperative, intraoperative, or postoperative information), wounds in areas other than below the ankle joint, and those diagnosed with tumors or psychiatric disorders.

**Fig. 1:**
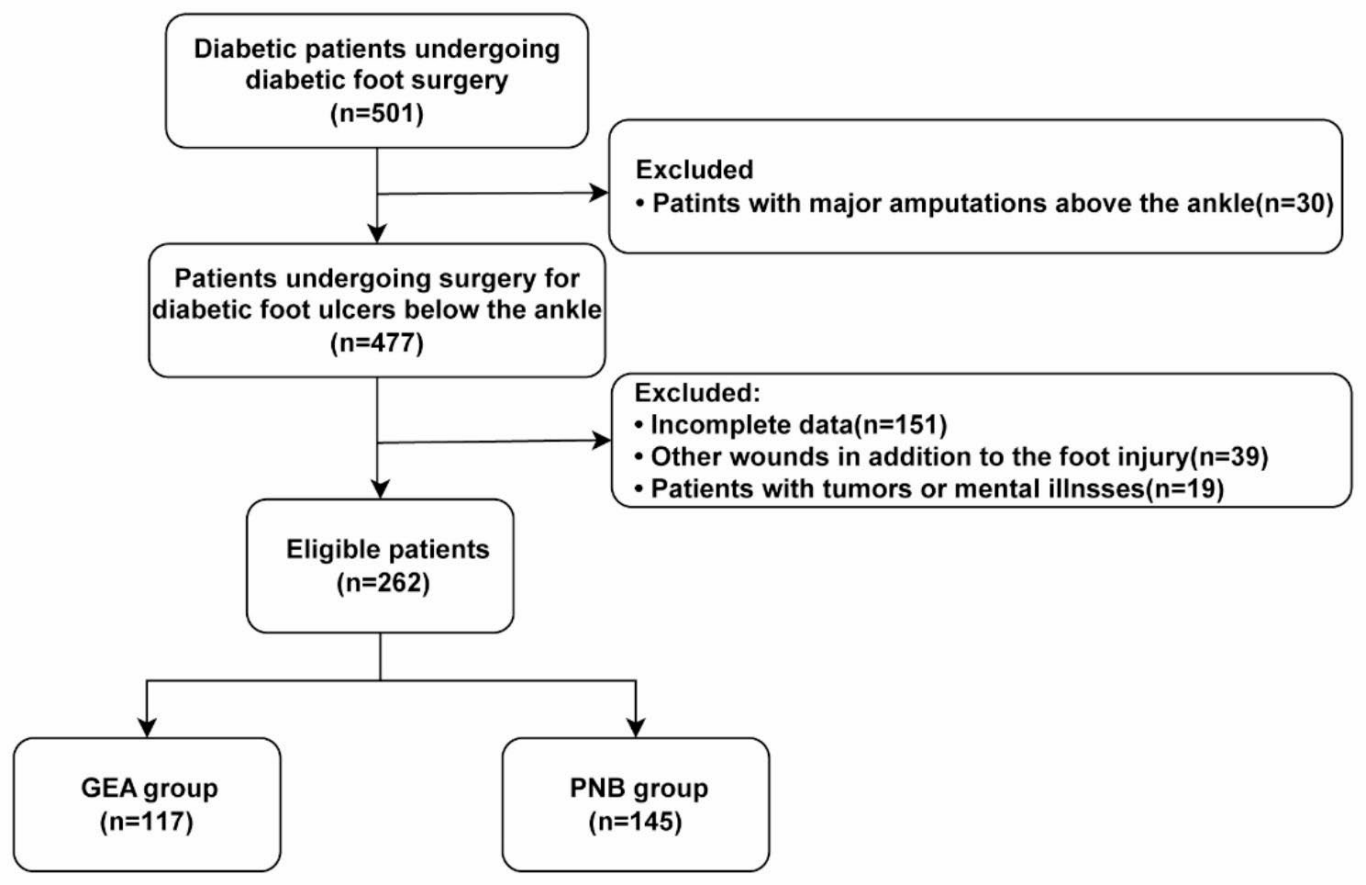
Participants’ screening flowchart

Initially, we selected all patients from our hospital’s electronic medical records system within seven years, from June 2015 to June 2022. Patients with diabetic foot were identified based on disease diagnosis codes, and we filtered outpatients who underwent surgery by examining surgical procedure codes, thus selecting patients with diabetic foot who had undergone surgical intervention. Subsequently, patients receiving GEA and ultrasound-guided PNB during surgery were chosen. This resulted in a total of 501 cases. In applying exclusion criteria, we first excluded 30 cases of severe amputation above the ankle joint during hospitalization. We then excluded 151 patients with incomplete clinical data, including 42 cases with incomplete preoperative information, 61 with incomplete intraoperative details, and 48 with incomplete postoperative information. Additionally, we excluded 39 patients with wounds in other areas besides below the ankle joint. Finally, 19 cases were excluded due to tumors (11 cases) or psychiatric disorders (8 cases). Ultimately, 262 patients met the inclusion criteria; then, the patients were divided into the GEA group and the PNB group based on their initial surgical anesthesia method. (Fig. 1).

### Anesthesia methods

In the GEA group, we induced anesthesia using sufentanil (0.2–0.5 µg/kg), etomidate (0.2–0.3 mg/kg), and cisatracurium besylate (0.10–0.15 mg/kg). Tracheal intubation was performed under laryngoscopy approximately 3 min later, followed by connection to a ventilator for mechanical ventilation. To maintain an end-tidal carbon dioxide level of 35–45 mmHg, a tidal volume of 6–8 mL/kg, a respiratory rate of 10–20 breaths/min, an inspiratory-to-expiratory ratio of 1:2, an oxygen concentration of 40% in the air, and positive end-expiratory pressure of 5 cmH_2_O were utilized for mechanical ventilation. Remifentanil (0.2–0.4 µg/kg/min) and propofol (4–8 mg/kg) were administered for GEA maintenance, with anesthesia depth adjustment based on the patient’s vital signs.

In the PNB group, we took ultrasound-guided sciatic nerve combined with a saphenous nerve block. Patients were positioned supine with the affected side up, maintaining an extended limb position on the affected side and a flexed position on the healthy side. Each patient underwent ultrasound scanning using a probe frequency of 6–13 MHz. The probe was placed above the popliteal fossa to visualize the transverse view of the sciatic nerve, appearing as an oval-shaped hyperechoic structure. The bifurcation point of the sciatic nerve and its junction with the tibial and common peroneal nerves were identified. Using ultrasound guidance, a needle was inserted from the lateral side, and a block was performed at the bifurcation site, injecting 20 mL of 4.5 g/L ropivacaine hydrochloride. Subsequently, with the patient supine, the probe was placed transversely at the 5 cm point above the medial aspect of the patella, gradually moving inward to visualize the obturator nerve beneath the fascia in the posterior middle part of the adductor canal. The ultrasound-guided obturator nerve block was injected with 20 mL of 4.5 g/L ropivacaine hydrochloride. Sensory and motor blockade were assessed after completing the anesthesia.

### Observation indicators

In this study, we have collected comprehensive preoperative, intraoperative, and postoperative metrics to evaluate the outcomes of diabetic foot ulcer surgery. The preoperative data included age, gender, weight, type of diabetes (type 1 or type 2), smoking status, ASA classification, patient complications, Charlson co-morbidity index, the disease history period, and Wagner grade for ulcer injury. Additionally, preoperative laboratory data such as white blood cells, red blood cells, hemoglobin, platelet count, creatinine, creatine kinase MB isoenzyme, D-dimer, prothrombin time, international normalized ratio, activated partial thromboplastin time, aspartate aminotransferase, total bilirubin, and albumin were recorded. We also noted the preoperative ASA classification, anesthetic type, and surgical procedure. In this study, we collected preoperative and postoperative laboratory data and statistically analyzed the differences between them. This analysis aimed to assess the impact of anesthesia on patients’ physiological conditions and provide insights into the effects of intraoperative anesthesia on systemic metabolic status. Moreover, it offered additional opportunities for data analysis and exploration. The collection of these data enhanced the comprehensiveness and accuracy of our understanding regarding the impact of anesthesia on patients with DFUs.

During the intraoperative phase, we recorded the anesthesia induction time, surgical time, anesthesia time, and fluid balance evaluation, including the volume of perioperative transfusions, intraoperative blood loss, and overall fluid input. We tracked significant hypotensive events defined as a decrease in systolic blood pressure of at least 30% from the pre-induction level, as these events are likely to compromise organ perfusion. During surgery, we routinely employed a cuff-based blood pressure monitoring method using a monitoring device to measure patients’ blood pressure. Additionally, we utilized electrocardiography equipment to monitor patients’ cardiac electrical activity and assess cardiac rhythm. The monitoring device automatically recorded intraoperative blood pressure and heart rate every 5 min. Mean blood pressure and heart rate were recorded before the start of the procedure (Pre-induction), 10 min after the beginning of the process (T1), 20 min (T2), 30 min (T3), 40 min (T4), 50 min (T5), 60 min (T6), and at the end of the procedure (End).

Postoperative data included pain ratings using NRS, the volume of anesthetic utilized in PCIA pumps for the first and second postoperative days, supplemental analgesic medications added within 24 h after surgery, time spent in the hospital, postoperative complications (pneumonia, nausea and vomiting, urinary retention), treatment results (0 = significant effect, 1 = effective, 2 = ineffective, 3 = death), and postoperative examinations. In treatment results, considerable improvement refers to a clear improvement in the postoperative ulcer condition. This is manifested by a reduction in ulcer size compared to admission, accelerated healing, and alleviation of symptoms. Effective denotes the successful control of the postoperative ulcer condition. Stable ulcer size, no further deterioration, and a certain degree of symptom relief characterize this. Ineffective indicates that the postoperative ulcer condition did not improve or worsen significantly. An increase in ulcer size, worsening of symptoms, and ineffective treatment, among other factors, characterize Ineffectiveness. Death refers to the mortality rate within 30 days post-surgery. The postoperative examinations included activated partial thromboplastin time, thrombin time, D-dimer, prothrombin time, international normalized ratio, total bilirubin, albumin, creatinine, creatine kinase MB isoenzyme, white blood cell and red blood cell counts, hemoglobin, hematocrit, and platelet count. These metrics were collected to assess the safety and efficacy of the surgical procedure and postoperative care.

Our study employed standard practices for postoperative pain management in patients undergoing surgery for DFUs. Both groups of patients received the same intravenous PCIA, which included 16 mg of ondansetron, 2 mg of butorphanol tartrate, and 75 mg of ketorolac tromethamine. The infusion rates and doses of analgesic medications were identical, set at two ml/h with a total volume of 100 ml. In addition to the PCIA, patients in our study also received intravenous infusion of analgesic medications. In addition to PCIA, our study also included intravenous administration of analgesic drugs. If the NRS score exceeded four or the patient requested additional analgesic medication, intravenous dezocine at 5 mg was administered. Therefore, we collected data on using supplemental analgesics within 24 h postoperatively.

Evaluate patients who have undergone a second postoperative surgery and amputation. We conducted follow-ups for all patients, and the follow-up outcomes were categorized as “death” and “censored data.“ The “censored data” category encompassed patients still alive, patients who could not attend scheduled follow-up visits, and patients who experienced death for reasons other than the condition under study. These data were used to assess 5-year mortality.

### Statistical analysis

In the present study, statistical analysis was conducted using SPSS version 26.0 and R software 4.2.2. The Shapiro-Wilk test was utilized to assess data normality. Means and standard deviations (SD) were reported for normally distributed continuous data, and independent-sample t-tests were performed to compare group means. The Mann-Whitney U test was employed for non-normally distributed continuous data and presented as medians (P25, P75). Categorical data were presented as frequencies, and their differences between groups were assessed using the chi-square or Fisher’s exact test (when applicable). Repeated measurements, such as NRS scores, MBP, and HR, were analyzed using linear mixed models. To evaluate the group-by-time interactions, changes over time between groups were compared. Survival analysis was performed using the Kaplan-Meier method. Statistical significance was set at a p-value of 0.05 or less.

## Results

In terms of baseline data for both groups of patients. Regarding age, when comparing the GEA group (62.93 ± 11.81) and the PNB group (64.98 ± 13.10), the P-value is 0.190, suggesting no significant difference and thus indicating comparability. There were no significant differences in age, gender, type of diabetes, smoking status, BMI, and the disease history period distribution between the GEA and PNB groups, with P-values all > 0.05 (Table 1).

**Table 1:** Characteristics of patients receiving PNB or GEA

	GEA(n = 117)	PNB(n = 145)	Statistical values	P value
Age(years)	62.93 ± 11.81	64.98 ± 13.10	t=-1.315	0.190
Gender			$\chi 2$=2.509	0.113
Male	82(70.1%)	88(60.7%)		
Female	35(29.9%)	57(39.3%)		
Type of diabetes				0.412
Type 1 diabetes	4(3.4%)	2(1.4%)		
Type 2 diabetes	113(96.6%)	143(98.6%)		
Smoking	39(33.3%)	48(33.1%)	$\chi 2$=0.002	0.969
Body Mass Index(kg/m2)	23.23 ± 2.55	23.35 ± 2.64	t=-0.393	0.694
CCI [M(P25,P75)]	2.60(2.00,4.97)	3.00(2.00,5.00)	Z=-1.663	0.960
DHP	30(15,64)	30(14,62.5)	Z=-0.305	0.760
Wagner			$\chi 2$=6.402	0.094
2	0(0.0%)	7(4.8%)		
3	64(54.7%)	74(51.0%)		
4	51(43.6%)	63(43.4%)		
5	2(1.7%)	1(0.7%)		
ASA classification			$\chi 2$=2.895	0.235
II	26(22.2%)	37(25.5%)		
III	84(71.8%)	105(72.4%)		
IV	7(6.0%)	3(2.1%)		

Values are presented as mean ± SD or the number of patients (%)

To assess the baseline comorbidity burden of the two patient groups, we used the Charlson Comorbidity Index (CCI), a commonly used scoring system to measure chronic disease burden in patients. It is based on a range of known diseases, assigning a certain weight to each disease to calculate the overall disease burden of the patient. These diseases include various conditions such as heart disease, diabetes, chronic kidney disease, malignant tumors, and others. Each disease is assigned a specific weight, and a total score is calculated based on whether the patient has these diseases and their severity. A higher score indicates a heavier disease burden and potentially poorer prognosis. In our study, when comparing the CCI of the GEA group [2.60 (2.00, 4.97)] and the PNB group [3.00 (2.00, 5.00)], the P-value is 0.960, indicating no significant difference and thus indicating comparability.

We also compared the interval between the onset of spontaneous disease and hospital admission for the two groups. Similarly, there was no difference between the GEA group [30 (15, 64)] and the PNB group [30 (14, 62.5)].

In assessing the admission wound, we used the Wagner classification for DFUs. Similarly, there were no differences between the two groups, indicating comparability.

Finally, we compared the preoperative American Society of Anesthesiologists (ASA) physical status classification for the two groups. The ASA classification system categorizes patients’ physical conditions to assess the risk level during surgery and anesthesia, enabling physicians to develop appropriate anesthesia and surgical plans. As observed, there was no difference between the two groups, indicating comparability. In summary, there were no differences in baseline characteristics between the GEA group and the ultrasound-guided PNB group, indicating comparability.

### Pre-operative situation

There was no significant difference in the period of disease history between the two groups of patients before surgery (Table 1). Table 2 compares the two groups regarding the duration of anesthesia maintenance, preparation time, and operation time. Our findings indicated no significant difference in anesthesia maintenance between the PNB and GEA groups (P > 0.05). Nonetheless, the preparation time in the PNB group was considerably longer than that in the GEA group (30 [20, 35] vs. 15 [10, 20], P < 0.05). Additionally, the operation time in the PNB group was notably more extended than that in the GEA group (P < 0.05).

**Table 2:** Comparison of pre-and post-treatment effect levels

Pre- and post-operative lab data difference	GEA(n = 92)	PNB(n = 108)	Statistical values	P value
PT	-0.3(-1.55,0.12)	-0.05(-0.68,1.03)	Z=-1.911	0.056
INR	-0.04(-0.15,0.02)	-0.01(-0.04,0.06)	Z=-1.856	0.089
APTT	-1.60(-2.80,1.15)	-0.10(-2.75,2.65)	Z=-0.904	0.366
FDP	0.95(0.31,2.11)	0.51(-0.39,1.08)	Z=-1.916	0.055
TT	0(-1.80,1.00)	-0.10(-1.35,0.58)	Z=-0.052	0.959
ALT	3(-1,6.5)	3(-1.95,4)	Z=-2.084	0.063
AST	2.98 ± 17.15	-0.03 ± 14.26	t = 1.530	0.127
TB	-0.2(-1.45,1.5)	-0.1(-1.55,1.4)	Z=-0.388	0.698
Albumin	0.4(-2.5,2.75)	0.6(-2.15,2.4)	Z=-0.322	0.748
Cr	0(-5.9,13)	0.1(-7.8,15.45)	Z=-1.579	0.114
CKMB	0(-3,2)	-0.1(-3,3.05)	Z=-0.118	0.906
WBC	0.92 ± 3.27	0.87 ± 2.98	t = 0.105	0.916
RBC	0.06(-0.22,0.27)	0.07(-0.11,0.315)	Z=-0.679	0.497
Hb	2(-5.5,7.5)	2(-3,8)	Z=-0.495	0.620
HCT	0.003(-0.023,0.019)	0.006(-0.010,0.024)	Z=-0.762	0.446
Plt, n (%)	12(-30,71)	16(-17,46.5)	Z=-0.752	0.452

Skewed data are described by median (interquartile spacing), and normal data are described by means ± SD

### Hemodynamic variables and heart rate

**Fig. 2:**
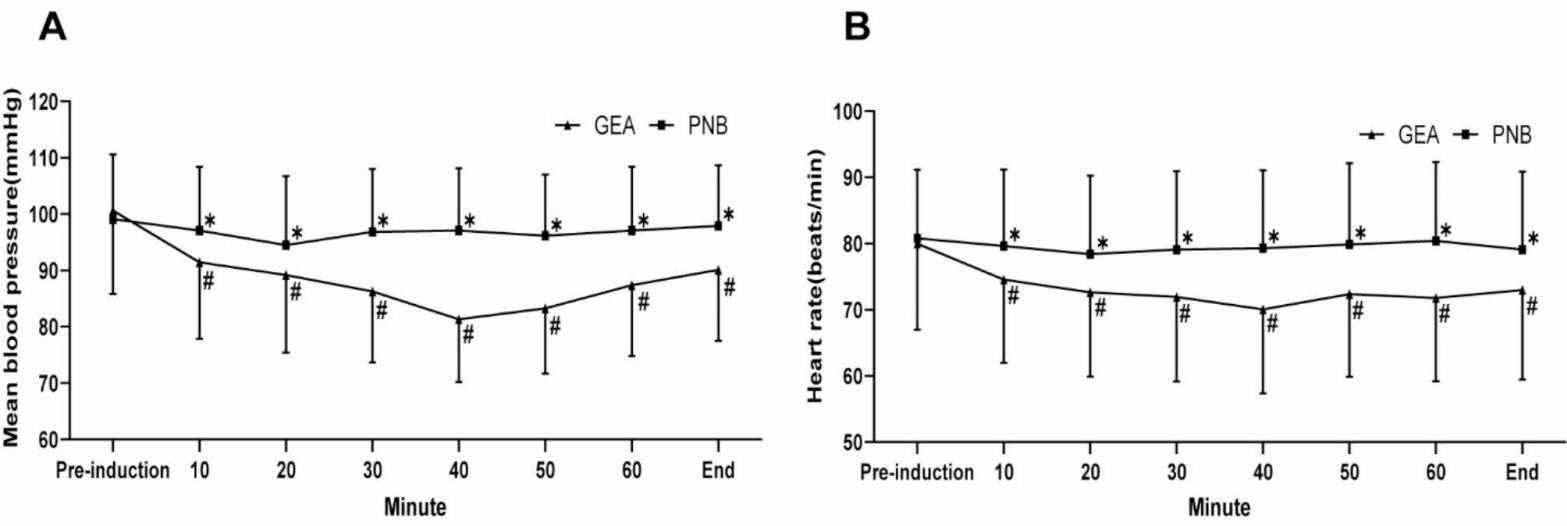
The mean blood pressure (**A**) and heart rate (**B**) during surgery

A linear mixed-effects model was utilized to analyze the effects of two anesthetic techniques on mean blood pressure and heart rate at various surgical time points. The results indicated a significant difference in the impact of the two anesthesia techniques on mean blood pressure and heart rate (P < 0.05). The PNB group exhibited more stable hemodynamics and heart rate than the GEA group, as illustrated in Fig. 2A and B. The mean blood pressure and heart rate varied significantly (P < 0.05) as the time points changed between the two anesthesia techniques. At T0, before surgery, there was no significant difference between the GEA and PNB groups regarding mean blood pressure and heart rate (P > 0.05). However, from T1 to T7, there was a significant difference (Bonferroni-corrected P 0.01) favoring the GEA group. The mean blood pressure of the GEA group was higher at T0 than at T1-T7 (P < 0.05). Moreover, Table 3 indicated no significant difference (P > 0.05) among any time points in the PNB group. Furthermore, significant differences (P < 0.05) were observed between the two groups regarding total fluid input, intraoperative blood loss, and perioperative blood transfusion. Table 4 also demonstrated a statistically significant difference between the GEA and PNB groups in the occurrence of severe hypotension during the operation.

Values are presented as means ± SD. (*) P < 0.05 versus the GEA group (Bonferroni corrected) ;(#) P < 0.05 versus the baseline value for each group. GEA = general anesthesia, OP = operation, PNB = popliteal nerve block, SD = standard deviation.

**Table 3:** Comparison of changes in hemodynamic indexes between the two groups at different periods (x ± s)

Indicators	Group	Pre-induction	T1	T2		T3		T4		T5		T6		End	
MBP (mmHg)	GEA (n = 145)	100.62 ± 14.81	89.19 ± 13.77∗	88.26 ± 12.61∗	82.31 ± 11.14∗#△◇		80.25 ± 11.57∗#△◇		87.34 ± 12.53∗○□◇		89.07 ± 12.57∗○□		91.45 ± 13.60∗		
	PNB (n = 117)	99.09 ± 11.50	97.07 ± 12.23	96.85 ± 11.16	97.05 ± 11.10		97.14 ± 10.88		97.07 ± 11.26		96.89 ± 10.75		97.07 ± 11.29		
	t	1.023	-5.558	-5.755	-9.861		-11.302		-6.507		-5.234		-3.760		
	P value	0.307	< 0.001	< 0.001	< 0.001		< 0.001		< 0.001		< 0.001		< 0.001		
	Between groups	F = 45.19, P < 0.001													
	Different points in time	F = 74.71, P < 0.001													
	Between groups • different time points	F = 51.86, P < 0.001													
HR (times/min)	GEA (n = 145)	79.98 ± 13.04	74.52 ± 12.56∗	72.63 ± 12.74∗	71.91 ± 12.77∗		70.05 ± 12.73∗#		72.36 ± 12.51∗		71.75 ± 12.56∗		72.95 ± 13.54∗		
	PNB (n = 117)	80.76 ± 10.35	79.60 ± 11.55	78.39 ± 11.84	79.09 ± 11.83		79.28 ± 11.78		79.84 ± 12.31		80.41 ± 11.92		79.06 ± 11.79		
	t	-0.512	-3.349	-3.795	-4.738		-6.083		-4.934		-5.707		-4.032		
	P value	0.609	0.001	< 0.001	< 0.001		< 0.001		< 0.001		< 0.001		< 0.001		
	Between groups	F = 20.60, P < 0.001													
	Different points in time	F = 27.74, P < 0.001													
	Between groups • different time points	F = 15.92, P < 0.001													

Note. ∗represents P < 0.05 compared with Pre-induction; #represents P < 0.05 compared with T1; △represents P < 0.05 compared with T2. ○represents P < 0.05 compared with T3. □represents P < 0.05 compared with T4. ◇represents P < 0.05 compared with End. Values are presented as mean ± SD.

**Table 4:** Intraoperative results

	GEA(n = 117)	PNB(n = 145)	Statistical values	P value
Preparation time, min	15(10,20)	30(20,35)	Z =-7.664	0.000
Operation time, min	50(30,69.25)	60(45,80)	Z =-4.207	0.000
Duration of Anesthesia, min	100(80,115)	100(79,110)	Z =-0.019	0.985
Total fluid input, ml	1100(1000,1200)	1100(600,1100)	Z =-4.813	0.000
Intraoperative blood loss, ml	50(50,100)	50(30,60)	Z =-3.142	0.002
Perioperative blood transfusion, ml	0(0,0)	0(0,0)	Z =-2.189	0.029
Significant hypotension (n, %)	16(13.7%)	3(2.1%)	$\chi 2$=12.968	0.000

Skewed data are described by median (interquartile spacing)

### Pain management

**Fig. 3:**
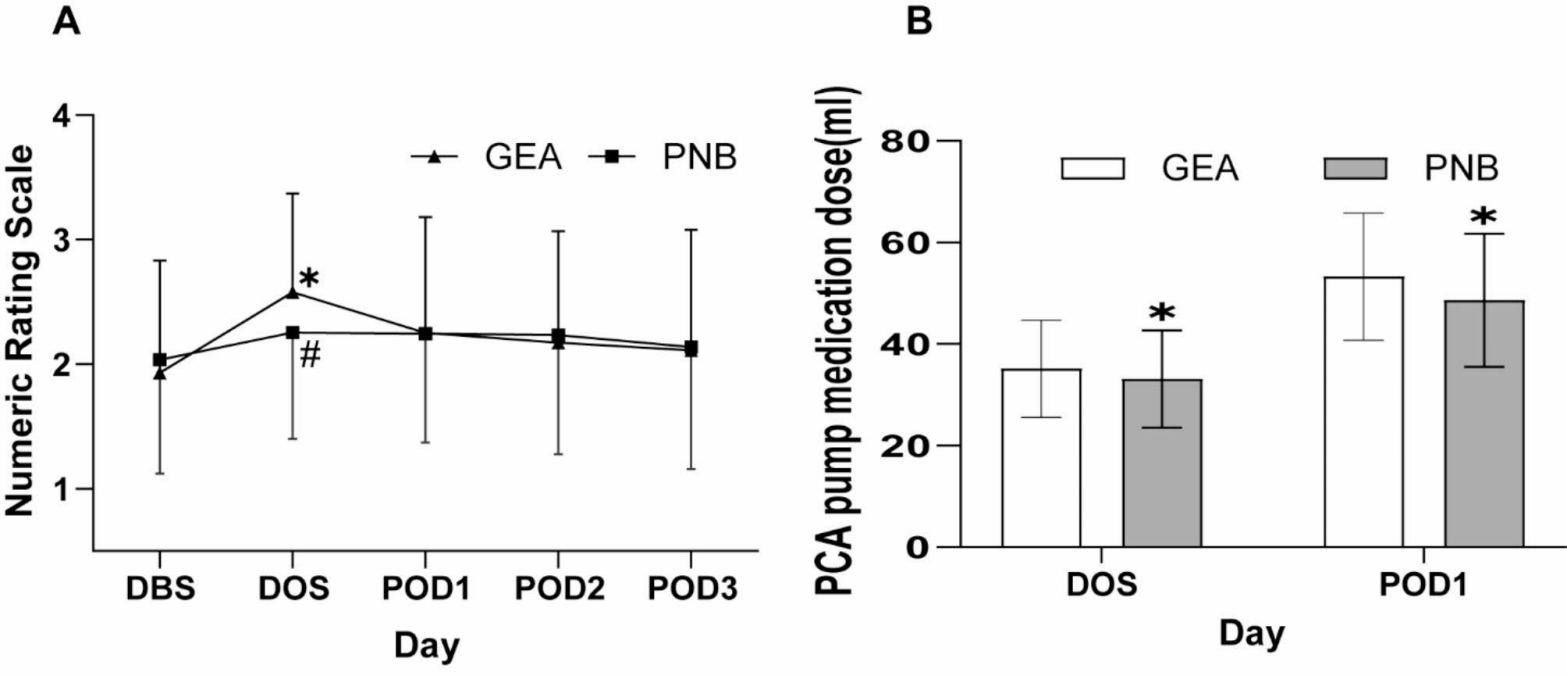
The NRS score was within one day before the surgery and three days after the surgery

In the present study, the NRS scores were analyzed to evaluate the efficacy of the two anesthesia techniques. The NRS scores obtained on the day before surgery did not differ significantly between the PNB and GEA groups (P > 0.05). However, as shown in Fig. 3A, the NRS score on the day of surgery was significantly higher in the GEA group compared to the PNB group (Bonferroni-corrected P 0.01). Moreover, PCIA requirements were substantially higher in the GEA group on the day of surgery than in the PNB group (P < 0.05), as depicted in Fig. 3B. The difference in the number of PCIA requirements remained significant even on the second day after surgery, with the GEA group requiring more PCIA than the PNB group (50(40,60) vs. 60(48,60), P < 0.05). Regarding the use of supplemental analgesics within 24 h postoperatively, the GEA group had a higher frequency than the PNB group (P = 0.018), indicating a statistically significant difference. (Table 5) These findings suggest that the PNB group had superior analgesic effects than the GEA group.

Values are presented as means ± SD. (*) P < 0.05 versus the GEA group (Bonferroni corrected) ;(#) P < 0.05 versus the baseline value for each group. GEA = general anesthesia, OP = operation, PNB = popliteal nerve block, SD = standard deviation.

**Table 5:** Postoperative results

	GEA(n = 117)	PNB(n = 145)	Statistical values	P value
Dezocine	14(12%)	6(4.1%)	$\chi 2$=5.627	0.018
Re-operation	106(90.6%)	130(89.7%)	$\chi 2$=0.064	0.800
Amputation	62(53.0%)	73(50.3%)	$\chi 2$=0.182	0.670
Hospitalization time, d	44(33,55.5)	45(29,60)	Z =-0.246	0.806
Treatment results			$\chi 2$=2.949	0.481
Significant effect	68(58.1%)	83(57.2%)		
Effective	30(25.6%)	47(32.4%)		
Ineffective	14(12.0%)	12(8.3%)		
Death	5(4.3%)	3(2.1%)		

Values are presented as the number of patients (%). Dezocine = Supplementary drug

### Prognosis

A comparison between two groups of postoperative complications showed that the incidence of postoperative pneumonia and nausea and vomiting in the PNB group was lower than in the GEA group, resulting in an overall significantly lower incidence in the PNB group (P < 0.05), as presented in Table 6. Of the 262 patients, 151 (57.6%) showed notable improvement, 77 (29.4%) were deemed effective, 26 (9.9%) were ineffective, and 8 (3.1%) died. Although the PNB group had a slightly shorter hospitalization time, the difference was not statistically significant. During hospitalization, there were five deaths in the GEA group and three in the PNB group, although the difference was not statistically significant. Table 5 showed no discernible difference in the treatment effectiveness between the two groups. According to the comparative results of the secondary surgery rates within 30 days of surgery between the two groups, both the GEA group (106 cases, accounting for 90.6% of the total sample) and the PNB group (130 cases, accounting for 89.7% of the total sample) exhibited relatively high rates of secondary surgeries. However, it is noteworthy that no significant differences were observed between the two groups in the statistical analysis. Similarly, in terms of the amputation rates within 30 days of surgery, we found no difference between the two groups regarding amputation and secondary surgery. Still, both groups had higher rates of secondary surgery and amputation. Please refer to Table 5 for specific data.

**Table 6:** Comparison of postoperative complications between the two groups (n, %)

	GEA (n = 117)	PNB (n = 145)	Statistical values	P value
Pneumonia	11(9.4%)	3(2.1%)	$\chi 2$=6.883	0.009
Nausea/vomiting	6(5.1%)	1(0.7%)		0.024
Urinary retention	3(2.6%)	1(0.7%)		0.327
The total incidence of adverse reactions	20(17.1%)	5(3.4%)	$\chi 2$=13.969	0.000

Values are presented as the number of patients (%)

**Fig. 4:**
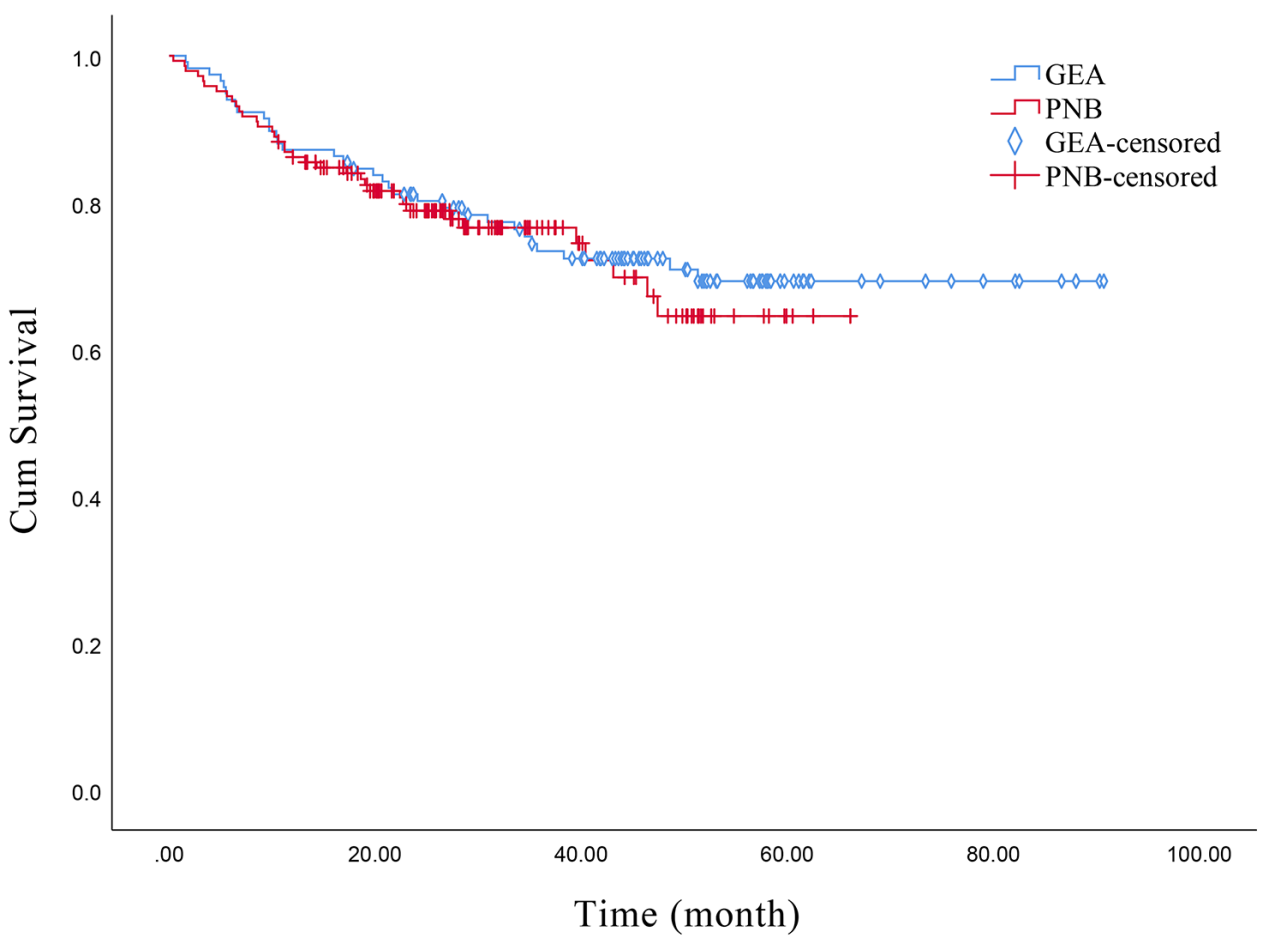
Survival Function

We followed up with all 262 patients in our study. Among them, 33 patients in the GEA group and 36 patients in the PNB group experienced death, while the remaining patients were classified as censored data. The category of censored data included 121 patients who were still alive, 55 patients who could not attend follow-up visits, and 17 patients who experienced death for reasons other than the condition under study. We conducted survival analysis for the two groups of patients and generated Kaplan-Meier curves. The log-rank test yielded a p-value of 0.666, while the Breslow test yielded a p-value of 0.750, indicating no significant difference in survival rates between the two groups. According to the Kaplan-Meier survival rate analysis, the 5-year mortality rate was calculated to be 32.5%. (Fig. 4)

## Discussion

The preparation time was significantly longer for the PNB group than for the GEA group (30 [20, 35] vs. 15 [10, 20] minutes, P < 0.05). The operative time was also different between the two groups, with the PNB group taking longer (60 (45, 80) vs. 50 (30, 69.25) minutes, P < 0.05). This disparity could be due to patients undergoing nerve blockades being awake and potentially moving their limbs during surgery, which could interfere with the surgeon’s work, or they may inquire about the status of the procedure, which could also interfere with the surgeon’s work. Moreira et al. [16] investigated the effects of various anesthetic techniques on the postoperative outcomes of elderly patients with partial or complete functional limitations who underwent significant lower limb amputation surgery. Similar to the findings of this study, they found that the different anesthetic techniques did not significantly impact patients’ postoperative outcomes. Still, the GEA group had a shorter operation time than PNB group. However, the length of anesthetic maintenance between the two groups was similar.

Regarding the intraoperative situation, the total fluid input was more in the GEA than in the PNB group. This may be related to the high incidence of hemodynamic instability and significant hypotensive events in the GEA group intraoperatively and the increased fluid input to maintain hemodynamic stability and improve hypotension. We found more perioperative blood transfusions in the GEA than in the PNB group. This finding is consistent with Malik et al. [17], who studied regional or spinal anesthesia for below-knee amputations to reduce the need for perioperative blood transfusions. A crucial part of patient care is reducing the usage of blood products due to the rise in healthcare expenses and the consequences of transfusions.

The results of the present study have provided evidence for several intraoperative and immediate postoperative advantages of PNB compared with the gold standard GEA. These observations largely align with earlier reports [18, 19]. The GEA group showed significantly lower intraoperative blood pressure than the PNB group, which could be attributed to the fact that the GEA group involves anesthesia and mechanical ventilation, which can exacerbate hypotension in already dehydrated patients, particularly those with poor physiological status, such as diabetic patients. On the other hand, PNB had a minimal effect on patients’ hemodynamic indices, suggesting that it significantly improved hemodynamic stability, greatly enhanced the safety of diabetic foot surgery, facilitated patients’ completion of the procedure, and positively influenced patients’ rapid postoperative recovery. Furthermore, it was observed that the incidence of intraoperative hypotensive events was significantly lower in the PNB group compared with the GEA group. Numerous studies have indicated that the incidence of postoperative acute kidney injury (AKI) is closely associated with intraoperative hypotension [20]. Specifically, the risk of AKI resulting from GEA was higher than that from nerve block anesthesia. This is thought to be partly because anesthetics employed during GEA have been found to trigger bouts of intraoperative hypotension [21]. A thorough investigation by Monk and colleagues revealed a positive correlation between intraoperative hypotension and heightened 30-day operative mortality rates [22]. This outcome could be attributed to ultrasound guidance during nerve block, which allows for precise targeting of nerve plexus anesthesia and significantly impacts intraoperative stress reduction while regulating the local anesthetic dosage. This approach enhances clinical efficacy and safety while actively suppressing patient stress reactions and reducing the likelihood of postoperative complications.

Comparing the PNB group to the GEA group, we discovered that the PNB group could keep intraoperative hemodynamic stability while successfully lowering postoperative pain in patients. This aligns with a prospective RCT experiment by Lai et al. that found PNB more effective at stabilizing hemodynamics and reducing postoperative pain. Compared to the GEA group, the PNB group maintained intraoperative hemodynamic stability while significantly reducing postoperative pain. Our findings are consistent with a prospective randomized controlled trial conducted by Lai et al. [23], which demonstrated that PNB was more effective than GEA at stabilizing hemodynamics and reducing postoperative pain. Using ultrasound equipment during nerve block, anesthesia may have contributed to this outcome by allowing for accurate localization and minimizing the risk of nerve damage, thereby promoting the reduction of postoperative pain; this may also mitigate the likelihood of chronic pain development [24]. Effective postoperative pain management improves patient recovery and reduces persistent pain [15, 18, 25]. Conversely, the GEA group’s use of the short-acting opioid Remifentanil may have contributed to the observed nociceptive hypersensitivity, potentially exacerbating postoperative pain [26–29].

Following anesthesia, several common adverse effects can occur. These may include the formation of postsurgical hematomas [30], instances of hypotension [21], as well as episodes of nausea and vomiting [31]. The incidence of complications in the PNB group was lower than that in the GEA group, possibly due to the precise nerve block placement under ultrasound guidance, which has a limited impact on the sympathetic nerves of the visceral organs and minimal effect on the patient’s physiological function. On the other hand, GEA involves intubation and other procedures that may compromise airway integrity, resulting in higher rates of postoperative pneumonia compared to the PNB group [20]. In terms of postoperative nausea and vomiting complications, there was a difference observed between GEA group [6 (5.1%)] and PNB group [1 (0.7%)]. This discrepancy may be attributed to various drugs in GEA, including anesthetics and muscle relaxants, which are known to affect the central nervous and gastrointestinal systems potentially. The deeper depth of GEA may stimulate the emetic center, increasing the risk of nausea and vomiting. Furthermore, GEA has the potential to inhibit gastrointestinal motility and gastric acid secretion, leading to delayed gastric emptying and retention of gastric contents, which further elevates the risk of postoperative nausea and vomiting. The factors above collectively contribute to the occurrence of nausea and vomiting. There was no difference in the rate of urinary retention complications between the two groups. These findings are consistent with previous studies [32], which have also demonstrated the safety and efficacy of ultrasound-guided PNB anesthesia in reducing postoperative complications.

The influence of different types of anesthesia on patient prognosis has been extensively studied in vascular surgery and other medical domains. However, definitive conclusions regarding whether anesthesia improves treatment outcomes remain elusive. Previous studies have shown that PNB for lower extremity vascular bypass can alleviate cardiovascular complications and reduce postoperative mortality in severe limb ischemia ulcers [33]. In contrast to bypass surgery, we found no significant difference in the effects of anesthesia type on recovery from the treatment of DFUs. This may be due to the fact that lower extremity vascular bypass surgery is generally more invasive and longer in duration than surgery for DFUs. And this study demonstrates that within 30 days postoperatively, both groups of patients had a relatively high re-intervention rate regarding secondary surgeries and amputations. However, the two groups were similar when comparing these rates.

Whether anesthesia modality influences mortality in DFUs remains a matter of debate, and our results suggest that the choice of anesthesia does not appear to have a substantial impact on patient mortality. In our study, we observed a 5-year mortality rate of 32.5%, consistent with the findings reported by Armstrong et al. [34] for diabetic foot ulcer patients (30.5%). However, our results differ from the study conducted by McDermott et al. [35], which suggested a higher 5-year mortality rate of 50–70% in diabetic foot ulcer patients. This discrepancy may be attributed to the higher number of patients lost to follow-up, potentially influencing the estimation of the 5-year mortality rate.

Several limitations of this study should be acknowledged. Firstly, the study results may be influenced by rules associated with retrospective data collection, including incomplete information retrieval, potential omissions, and selection biases. Furthermore, due to the absence of randomization, we cannot exclude the impact of treatment preference on the results, which could introduce biases. To minimize the influence of these limitations, we implemented detailed data collection and statistical analysis measures in the study. Secondly, considering that this is a single-center investigation with a relatively small sample size and also subject to the constraints of retrospective research, the generalizability of the study findings may be affected, and we might need to identify significant correlations. Further research with more extensive multi-center studies is required to validate these findings and enhance the reliability of the results. Lastly, our study is observational, implying that individual physician judgment could still influence the selection of anesthesia techniques. Therefore, when interpreting these study results, we must carefully consider the potential impact of unique variations and other underlying factors.

## Conclusions

To conclude, our study highlights the effectiveness of ultrasound-guided PNB anesthesia in enhancing clinical safety during the surgical treatment of DFUs. By providing precise localization of nerve blocks, ultrasound guidance notably reduces the total volume of fluid required during surgery. This, in turn, promotes hemodynamic stability, lowers postoperative pain levels (as assessed by NRS scores), and diminishes the reliance on analgesic medications. Although the 5-year mortality rate is 30.5%, no significant difference in mortality rates between the two groups was observed. These findings underscore the clinical significance and practicality of employing ultrasound-guided PNB anesthesia in the surgical management of DFUs.

### Electronic supplementary material

Below is the link to the electronic supplementary material.

STROBE Statement?checklist of items that should be included in reports of observational studies

## Data Availability

The publicly available datasets used in this study can be found in the First Affiliated Hospital case system of Nanchang University, Jiangxi Province, China.
